# Students’ knowledge, perceived access, and attitudes toward population health integration in undergraduate nursing education

**DOI:** 10.3389/fpubh.2026.1898158

**Published:** 2026-07-17

**Authors:** Liangzhe Wang, Jun Wang

**Affiliations:** School of Medicine, Wuhan University of Science and Technology, Wuhan, China

**Keywords:** attitudes, integration, knowledge, perceived access, population health, undergraduate nursing education

## Abstract

**Background:**

To address the growing population health challenges, the nursing workforce is expected to possess competencies of population health, which has been officially included as a new essential component in nursing and strongly advocated by nurse educators worldwide. However, no previous studies have explored the relevant opinions among nursing students, who act as recipients, beneficiaries, and key stakeholders in nursing education. This study aimed to explore students’ population health knowledge, perceived access to relevant training, and attitudes toward integrating population health content within undergraduate nursing curricula.

**Methods:**

A cross-sectional observational survey was conducted using a self-developed questionnaire among nursing students from a Chinese university in January 2026. Data collected included demographic information, population health knowledge levels measured via both self-assessment and objective evaluation, perceived accessibility of undergraduate training related to population health competencies, attitudes toward integrating population health into undergraduate nursing education, and preferred integration models.

**Results:**

A total of 163 nursing students who provided valid responses were included in the final analysis. The mean self-assessed and objective scores of population health knowledge (rated on a 5-point scale) were 2.98 ± 0.10 and 0.71 ± 0.06, respectively. Less than 20% of participants reported having frequent or very frequent access to educational opportunities targeting core population health competencies. Nearly half of the students agreed or strongly agreed with the importance of population health and its inclusion in nursing education, particularly at the undergraduate level, and 73.0% expressed their willingness to systematically learn about population health. Furthermore, 44.2% students believed that population health should be longitudinally integrated into the current undergraduate nursing education framework, while 21.5% preferred the “lecture-based elective course” as pedagogical model.

**Conclusion:**

Findings indicate insufficient preparedness in population health competencies among future nursing professionals. Integration of population health into undergraduate nursing education is necessary and generally acceptable to students.

## 1 Introduction

The strong association between individual and population health outcomes, evidenced by the fact that less than 15% of health outcomes stem from medical care, with the majority influenced by environmental exposures, social circumstances, behavioral patterns, and genetics, has long been recognized (1, 2). First articulated by Kindig and Stoddart (3), this association is encapsulated in the concept of population health, defined as “*the health outcomes of a group of individuals, including the distribution of such outcomes within the group*.” As a broad term encompassing prevention, health inequalities, healthcare delivery systems, policy-making, risk factors and social determinants of health, *etc*., population health focused on the aggregate or population dimension, rather than individual healthcare, with an emphasis on analyzing health determinants and outcomes within specific groups and using population health-level data to guide targeted policies and interventions linking these two domains (1–8).

Over the past decades, global health systems face mounting population health threats, including population aging, rising healthcare expenditures, growing burdens of chronic and mental illnesses, and frequent outbreaks of epidemic diseases. These crises expose limitations of traditional individual-focused clinical care models and threaten system sustainability (9, 10). To address these challenges and improve population-wide health, the nursing workforce, as core frontline healthcare providers, is expected to transform into “new nurses” well-versed in population health domains, including epidemiology, disease prevention, lifestyle and nutrition interventions, social determinants of health, healthcare economics, health equity, and policy, *etc*., beyond their traditional illness-centered clinical expertise (5, 7, 11–13). Equipped with such knowledge, such “new nurses” can mitigate population health disparities and address multifaceted community health risks. The COVID-19 pandemic further underscored the critical gap between hospital-based clinical nursing and population health practice (5, 13–17).

Currently, public health nursing is a mature specialty worldwide that delivers community-centered, population-focused disease prevention and health promotion services (18, 19). Although universally recognized practical frameworks remain lacking (20), public health nurses serve as community public health leaders, responding to population aging, climate hazards, emerging pathogens, and epidemics of obesity and substance use to meet home- and community-based population health demands (18, 19). Even within hospitals and outpatient clinics, combining clinical judgment with population health perspectives optimizes patient care and is recognized as a core component of contemporary nursing practice (14, 21, 22). Existing evidence verifies that nurse-led population health interventions strengthen patients’ health awareness, self-efficacy, disease self-management and medication adherence across conditions such as hepatitis C, hypertension and asthma (23–26). Hospital-based nursing resources and workforce also form an essential backbone of population health emergency response for epidemics, disasters and public safety incidents (27, 28).

To provide comprehensive healthcare services to populations and patients in both community and clinical settings, nurses need to possess population health competencies across a spectrum of disciplines, such as environmental health, social justice, cultural competency, epidemiology, health policy, demography, disaster emergency, public health biology, population projections, and population behavioral sciences. This creates an urgent mandate for modern nursing education reform (7, 28–30). The value of population health training for nurses is widely acknowledged, yet most relevant educational opportunities target licensed nurses via continuing education, postgraduate master’s and doctoral tracks (12, 20, 31–34). In the United States (U. S.), specialized programs embedded in specialized graduate degrees, including Master of Public Health Nursing, Master of Population Health Nursing, post-Master Doctor of Nursing Practice in Public/Population Health Nursing, or Master of Public Health (MPH), are designed to prepare bachelor’s-level nurses for advanced population health roles (12, 32–35). In many countries around the world, advanced practice nurses (APNs) or registered nurses (RNs) may select population health as their core professional focus (12, 20, 33). Notably, the American Association of Colleges of Nursing (AACN) has incorporated Population Health as a core competency for APNs in its Core Competencies for Professional Nursing Education in 2021 (36).

At the undergraduate education stage, despite growing calls to embed population health into nursing curricula (5), empirical data over the past decade reveal persistent deficits, including fewer qualified faculty specialized in population health nursing, and insufficient undergraduate training on population health content (5, 13, 22, 37, 38). Barriers at institutional and clinical levels include the broad scope of population health topics without consistent conceptual frameworks, shortages of experienced instructors, limited educational budgets, and insufficient community practice placements for undergraduates (5, 13, 22, 37, 38). Accordingly, targeted improvements are urgently needed to advance population health integration within pre-registration nursing undergraduate programs.

As primary recipients, beneficiaries and core stakeholders of undergraduate nursing education, nursing students’ demands and preferences directly shape the effectiveness of population health curriculum integration. Their perspectives must therefore be prioritized in educational reform planning. The present study adopted a student-centered perspective to investigate Chinese nursing students’ population health knowledge, perceived access to relevant training, and attitudes toward embedding population health content in nursing curricula. The results may guide nursing educators to develop tailored training strategies and cultivate nursing graduates capable of addressing diverse population health needs and delivering high-quality integrated healthcare.

## 2 Methods

### 2.1 Study description

This cross-sectional observational survey was conducted in January 2026 using a self-developed questionnaire. All undergraduate and graduate nursing students enrolled at Wuhan University of Science and Technology, a comprehensive university in Hubei Province, central China, were invited to participate. The university offers four-year undergraduate nursing programs and nursing master’s degree tracks.

The study protocol and final questionnaire were approved by the Ethics Committee of Medicine College, Wuhan University of Science and Technology (Approval No. 2025H869). Prior to receiving the questionnaires, all participants read and signed an electronic informed consent form clarifying voluntary participation and full response confidentiality.

### 2.2 Questionnaire

The preliminary questionnaire was developed with reference to existing population health literature (1–8) and studies on population health integration within nursing education (12, 20, 31–38). Initially formulated in English, the questionnaire was translated into Chinese for administration. Two independent experts with expertise in nursing education and population health separately evaluated the questionnaire’s content validity. To ensure the reliability, conciseness, and relevance of the items, a pretest was conducted on a convenience sample of 15 nursing students, who were excluded from the formal survey.

Following minor revisions based on feedback and reliability-validity analysis of the pretest data, the questionnaire was finalized. The final questionnaire comprised 22 structured items divided into four sections: Demographic Information, Population Health Knowledge, Perceived Access to Population Health Education, and Attitudes Toward Population Health Integration in Undergraduate Nursing Curricula. The Demographic Information section contained three items (Q1–Q3) collecting basic background data including gender, age, and academic grade. The Population Health Knowledge section included one question item based on 5-point Likert scale (Q4) for self-assessed population health understanding, as well as 5 single-choice questions (Q5-9) for objective knowledge evaluation. The objective test covered the following aspects: the concept (Q5) and core objectives (Q6) of population health, the definition of “population” in the context of population health (Q7), the relationship between population health and public health (Q8), and the subtopics in population health (Q9). One point was awarded for each correct single-choice response, with a maximum objective knowledge score of 5. The Perceived Access section (Q10-15) asked students to rate their exposure to six AACN-defined population health core competencies (36) throughout undergraduate training on a 5-point Likert scale (1 = never; 2 = very infrequently; 3 = occasionally; 4 = often/frequently; 5 = very frequently). In the fourth section for Attitudes, four initial 5-point Likert items (1 = strongly disagree to 5 = strongly agree) were used to measure opinions on population health competency requirements for clinical nurses (Q16), the importance of integrating population health in nursing training (Q17), and the necessity of population health training for undergraduates (Q18). Respondents who selected “disagree,” “strongly disagree,” or “undecided” in response to Q18 were required to select their primary reason for disagreement (Q19). An additional 5-point Likert item (Q20) was designed to measure students’ willingness to learn population health content. Furthermore, two single-choice items in the Attitude section (Q21, Q22) investigated preferred curriculum integration models. The translated questionnaire is provided in the Supplementary material.

The Cronbach’s *α* coefficient of the finalized questionnaire was 0.82, and the Kaiser-Meyer-Olkin (KMO) measure yielded a value of 0.82.

### 2.3 Data collection

The online questionnaire link was disseminated to all nursing undergraduates and postgraduates via multiple recruitment channels. Undergraduates received the link through official class WeChat groups, with additional offline classroom promotion during teaching hours. Postgraduate students were reached via academic supervisors, course instructors and grade-level student unions to ensure full cohort coverage. During the entire data collection period, research team members consistently promoted the study and recruited voluntary participants offline and online. Prior to survey initiation, all potential respondents were fully apprised of the research objectives, and electronic informed consent was obtained from each participant prior to their engagement in the study. To minimize response bias arising from over-deliberation, distraction, or peer consultation, participants were instructed to complete the questionnaire within 15 min. The survey was voluntary and anonymous, and only fully completed questionnaires were included in the final data analysis.

None of the research team members were responsible for the teaching, assessment, or academic administration of the participating nursing students, eliminating potential implicit pressure and participation bias stemming from teacher–student relationships. All participants completed the questionnaire after finishing relevant coursework and receiving final grades. This timeline avoided confounding influences from ongoing classes or pending assessments, securing authentic and unbiased subjective feedback.

### 2.4 Statistical analysis

All questionnaire-derived data were imported into IBM SPSS Statistics version 26.0 for statistical computation. Categorical variables were expressed as frequencies (percentages), and intergroup comparisons across subgroups stratified by demographic characteristics were conducted using the chi-square (*χ*^2^) test or Fisher’s exact test. Quantitative variables were reported as mean± standard error of the mean (SEM). For between-group comparisons of knowledge scores as well as Likert-type perceived access and attitude scores stratified by demographic variables, independent samples t-tests were employed for pairwise comparisons, while one-way analysis of variance (ANOVA) followed by Tukey’s honestly significant difference *post hoc* test was applied for multiple group comparisons. Statistical significance was defined as a two-tailed *p*-value < 0.05.

## 3 Results

### 3.1 Demographic characteristics of nursing student respondents

A total of 189 nursing students at the target university were recruited, of whom 170 completed and returned the questionnaires, corresponding to an overall response rate of 89.9%. After excluding 7 questionnaires for failing to meet the 15-min completion requirement, 163 valid responses were included for final analysis. Detailed demographic characteristics of the participants are summarized in Table 1. Of the 163 eligible participants, the majority were female (*n* = 103, 63.2%). The mean age of the cohort was 21.0 ± 0.2 years. With respect to academic grade level, 28.2, 20.9, 19.6, and 15.3% of participants were first-, second-, third-, and fourth-year undergraduates, respectively, while the remaining 16.0% were nursing postgraduates.

**Table 1:** Demographic information and knowledge score of nursing students participating in the survey (*n* = 163).

Participant attribute	Number (%)	Mean ± SEM of self-assessed knowledge score	*p* value	Mean ± SEM of objectively measured knowledge score	*p* value
Gender					
Male	60 (36.8)	3.35 ± 0.18	0.003**	0.80 ± 0.09	0.119
Female	103 (63.2)	2.76 ± 0.12		0.66 ± 0.07	
Age					
≥21 years old	83 (50.9)	3.01 ± 0.16	0.360	0.76 ± 0.08	0.199
<21 years old	80 (49.1)	2.94 ± 0.14		0.66 ± 0.09	
Academic grade					
1 st-year undergraduate	46 (28.2)	3.02 ± 0.17	0.453	0.70 ± 0.12	0.590
2 nd-year undergraduate	34 (20.9)	3.03 ± 0.23		0.68 ± 0.13	
3 rd-year undergraduate	32 (19.6)	2.97 ± 0.25		0.84 ± 0.13	
4 th-year undergraduate	25 (15.3)	2.96 ± 0.30		0.64 ± 0.14	
Postgraduate	26 (16.0)	2.85 ± 0.26		0.69 ± 0.13	
Total	163 (100)	2.98 ± 0.10		0.71 ± 0.06	

**p < 0.01.

### 3.2 Population health knowledge

As presented in Table 1, the mean self-assessed score of the 163 respondents on population health was 2.98 ± 0.10 on a 5-point Likert scale. However, their actual scores measured through objective knowledge test yielded a mean of 0.71 ± 0.06 out of 5, indicating that the participants’ actual knowledge of population health was extremely low. Specifically, the correct response rates for the concept of population health (Q5), its core objectives (Q6), and the definition of “population” in the context of population health (Q7) were 6.8% (*n* = 11), 22.1% (*n* = 36), and 22.7% (*n* = 37), respectively. In particular, regarding the relationship between population health and public health (Q8), only 19 participants (11.7%) correctly selected the option stating that “*population health and public health are two distinct yet interconnected disciplines*,” whereas a predominant majority (*n* = 107, 65.6%) held the misconception that “*population health is synonymous with public health, serving as an alternative designation for the latter*.” Furthermore, when asked about “*which of the following is NOT a subtopic of population health*” (Q9), only 13 participants (8.0%) accurately identified the distractor “*incidence of outpatient visits and hospitalizations.*” Stratified analyses by demographic characteristics revealed a statistically significant difference in self-assessed knowledge scores between gender groups (*p* < 0.01), with male participants exhibiting greater self-confidence in their population health knowledge. However, no significant differences were detected in the objective knowledge scores across all participant subgroups (*p* > 0.05).

### 3.3 Perceived access to undergraduate population health competency training

**Figure 1:**
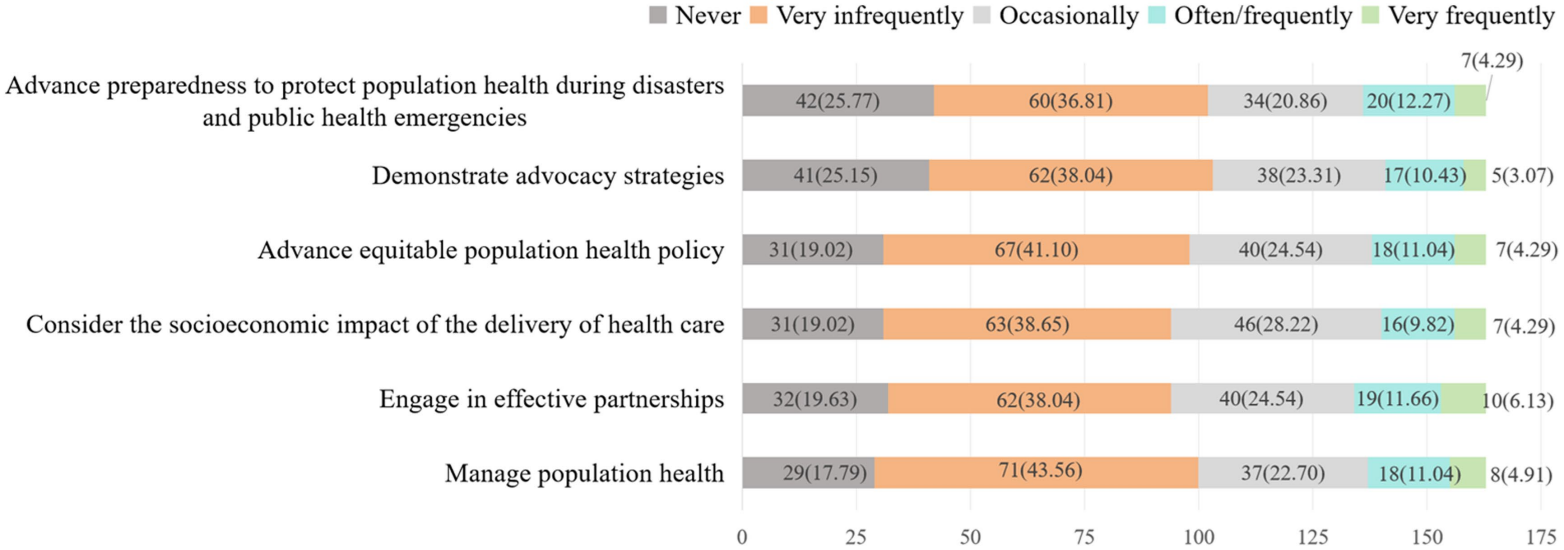
Self-perceived access to population health competency-related educational opportunities among the responding nursing students (*n* = 163). Data are presented as frequencies (percentages).

Figure 1 summarizes students’ perceived exposure to training covering six AACN-defined core population health competencies (36): manage population health, engage in effective partnerships, consider the socioeconomic impact of the delivery of health care, advance equitable population health policy, demonstrate advocacy strategies, advance preparedness to protect population health during disasters and public health emergencies. Although individual perceptions varied, fewer than 20% of students reported frequent or very frequent exposure to relevant undergraduate teaching. Group comparisons showed perceived training access was not significantly associated with gender, age or academic year (all *p* > 0.05).

### 3.4 Attitudes toward population health integration in undergraduate nursing curricula

**Figure 2:**
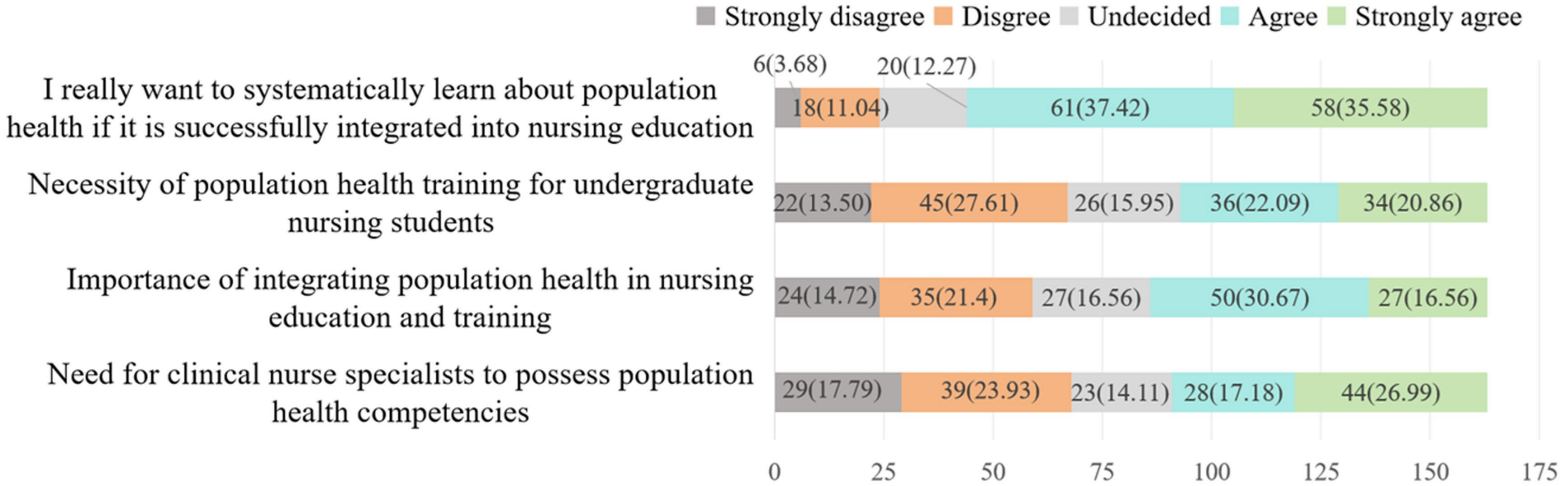
Attitudes of the responding nursing students toward integrating population health in nursing education (*n* = 163). Data are presented as frequencies (percentages).

Responses to the first three attitudinal Likert items were dispersed (Figure 2). In total, 42.9, 47.2, and 44.2% of the responding nursing students agreed or strongly agreed the need for clinical nurse specialists to possess population health competencies (Q16), the importance of integrating population health in nursing education and training (Q17), as well as the necessity of population health training for undergraduate nursing students (Q18), respectively. These findings indicate that, in general, the nursing students held positive perceptions regarding integrating population health into nursing education.

**Figure 3:**
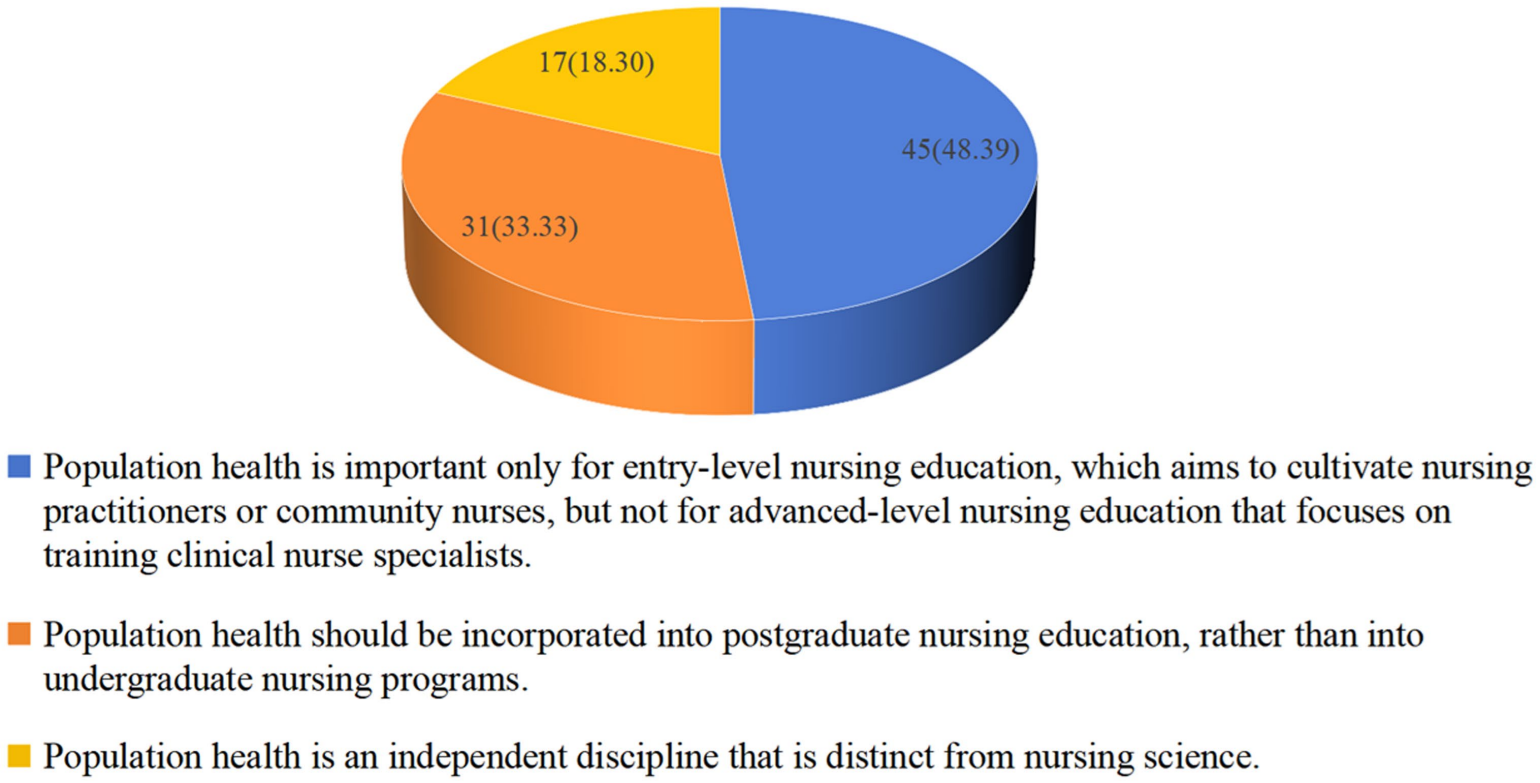
The most important reason perceived by students for their disagreement with the necessity of population health training for undergraduate nursing students (*n* = 93). Data are presented as frequencies (percentages).

Figure 3 displays responses from the 93 students who disagreed, strongly disagreed, or felt undecided regarding the necessity of population health training for undergraduate nursing students. Nearly half believed that “*Population health is important only for entry-level nursing education, which aims to cultivate nursing practitioners or community nurses, but not for advanced-level nursing education that focuses on training clinical nurse specialists*;” the other one-third had the opinion that “*Population health should be incorporated into postgraduate nursing education, rather than into undergraduate nursing programs*.” Encouragingly, an overwhelming majority (73.0%) of the respondents claimed that they really want to systematically learn about population health if it is successfully integrated into nursing education (Q20).

We found that responses to these four Attitudes questions assessed using a 5-point Likert scale were significantly associated with gender and age. Specifically, male students and older students exhibited more positive attitudes toward the integration of population health into nursing education compared with their female and younger counterparts, respectively. Detailed statistical results are as follows: (Q16) females: 2.89 ± 0.15, males: 3.53 ± 0.18, *p* = 0.006; students aged < 21 years: 2.79 ± 0.16, students aged ≥ 21 years: 3.43 ± 0.16, *p* = 0.003; (Q17) females: 2.94 ± 0.14, males: 3.48 ± 0.14, *p* = 0.009; students aged < 21 years: 2.91 ± 0.15, students aged ≥ 21 years: 3.34 ± 0.14, *p* = 0.021; (Q18) females: 2.87 ± 0.14, males: 3.49 ± 0.15, *p* = 0.004; students aged < 21 years: 2.85 ± 0.16, students aged ≥ 21 years: 3.33 ± 0.14, *p* = 0.013.

### 3.5 Preferred population health curriculum integration models

**Figure 4:**
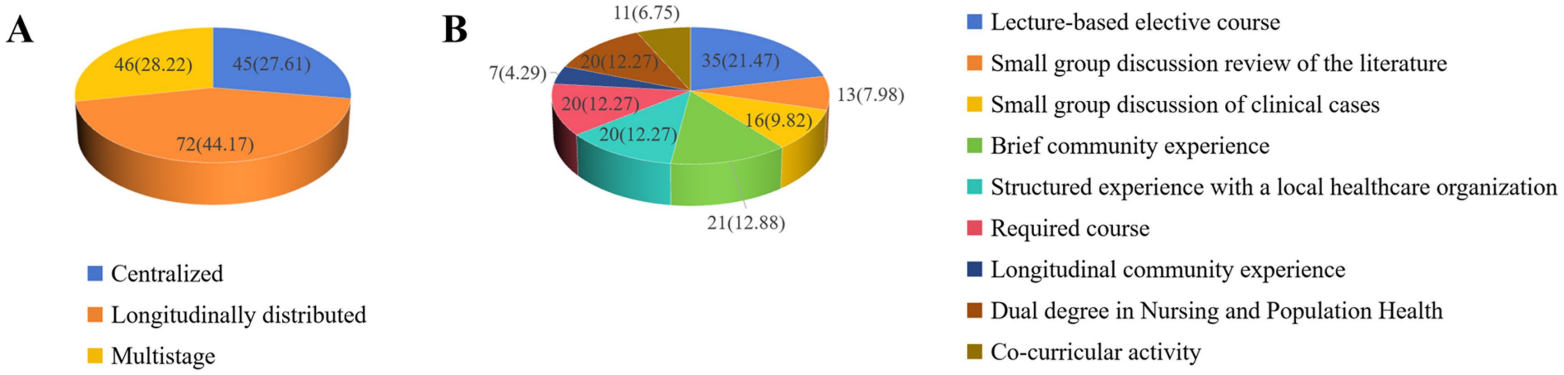
Students’ preferred mode **(A)** and teaching model **(B)** for integrating population health into the current undergraduate nursing education framework (*n* = 163). Data are presented as frequencies (percentages).

Figure 4 illustrates students’ preferred formats for embedding population health content in current undergraduate nursing education framework. In terms of the preferred mode of integrating population health into undergraduate nursing education, most students (44.2%) considered that population health should be *longitudinally distributed* throughout the current undergraduate nursing education framework (Figure 4A). Among the varied pedagogical models for delivering content related to population health, despite scattered responses, the top five most popular models were “*lecture-based elective course*,” “*brief community experience*,” “*structured experience with a local healthcare organization*,” “*lecture-based required course*,” and “*dual degree in Nursing and Population Health*,” which were chosen by 21.5, 12.9, 12.3, 12.3, and 12.3% of the responding nursing students, respectively (Figure 4B). The Chi-square test or Fisher’s exact test showed no significant differences in students’ preferred mode and teaching model for integrating population health into undergraduate nursing education by gender, age, or academic grade (all *p* > 0.05).

## 4 Discussion

With the advancement of public health science, growing forces have steered public health away from its historical mission of “*assuring the conditions in which people can achieve health*.” To date, public health has been closely tied to its more influential counterpart, medicine, thus remaining too biomedical and focused on personal or individual services. Over-reliance on randomized controlled trials, insufficient theoretical frameworks, and a fear of politics have also diverted the focus of the public health field (6, 8, 39). To address these constraints, population health science has emerged with its own theoretical commitment: *the health and health equity of a population are distinct from and determined differently from the health of individuals*. In particular, the term “population health” has been increasingly adopted in the nursing field (5, 7, 11–13), marked by the AACN’s 2021 designation of population health as a mandatory core nursing competency (36). To our knowledge, the present study is the first to investigate the students’ population health knowledge, perceived training access, and attitudes toward curriculum integration.

Chinese undergraduate nursing curricula follow a discipline-centered medical framework originating from the 1910 Flexner Report. Training prioritizes advanced clinical nursing skills, and most graduates work as clinical specialists focused on individual patient care or high-risk subgroups (40, 41). Especially in recent years, the orientation of nursing education in China has gradually shifted toward enabling nurses to pursue higher-level clinically focused degrees (40, 41). Currently, national undergraduate nursing accreditation standards in China do not mandate formal public health nursing coursework (42). However, COVID-19 has driven nursing educators to gradually incorporate public health content into classroom teaching (42, 43). Compared with public health, population health remains an unfamiliar emerging concept for Chinese nursing students. Our data showed a mean self-rated population health knowledge score of 2.98 out of 5, indicating moderate yet insufficient self-perceived understanding. To avoid self-report bias, we supplemented five objective single-choice knowledge items; participants averaged merely 0.71 out of 5 on this objective assessment, far below their self-reported confidence. Interestingly, an overwhelming majority of the responding students incorrectly believed that “*population health is synonymous with public health*.” Accordingly, it can be speculated that many respondents’ self-perceived scores were incorrectly based on their understanding of public health rather than population health. Subgroup analyses revealed male students reported higher self-confidence in population health knowledge, yet objective test scores showed no gender differences. This pattern aligns with documented gender gaps in academic self-evaluation and response bias (44). Additionally, although not statistically significant, there appears to be a decreasing trend in self-assessed knowledge across academic years. At the studied university, junior nursing undergraduates receive introductory public health courses that may build positive but superficial self-confidence, while senior students predominantly focus on specialized clinical courses with limited population health exposure, which may gradually diminish their perceived proficiency in this field. Meanwhile, objectively measured knowledge appears somewhat higher among third-year students, which may be due to curriculum structures. The third academic year is a critical transitional stage with intensive professional coursework that integrate public health and pre-clinical training, where certain courses may optionally deliver learning opportunities related to population health. In contrast, freshmen have not yet received systematic relevant training, and senior students prioritize clinical internships, resulting in decreased population health knowledge retention. These trends reveal the fragmented, stage-imbalanced nature of current population health education in undergraduate nursing curricula, providing practical insights for staged curriculum optimization and targeted teaching reform.

Students’ limited population health knowledge corresponded directly with their scarce exposure to competency-focused teaching. The 2021 AACN guideline establishes six standardized population health core competencies for nursing education (36). As a pioneering and authoritative guideline in this field, the AACN framework has been widely recognized by global nursing educators and academic communities as an indispensable component of modern nursing education (5, 22, 44). However, substantial gaps persist in its practical implementation worldwide. Even in the U. S., a pioneer in population health nursing education, national surveys revealed that while 88–89% of nursing educators intended to integrate population health content into routine teaching, most institutions failed to provide targeted faculty training and developmental programs to support competency-oriented instruction (5). Another survey showed that, of the12 programs provided by 96 identified nursing schools in the U. S., only 3 programs involving 5 nursing schools had included population health competencies. Of these, one offered Master of Science in Nursing (MSN) or MSN/Master of Business Administration (MBA) programs, and two were Doctor of Nursing Practice (DNP) and DNP/MBA partnership programs, highlighting a severe shortage of population health learning opportunities for undergraduate nursing students (45). In non-U. S. regions, empirical evidence regarding the integration of population health competencies into nursing education remains scarce. Donaghy et al. (38) reported that only 26% of universities in the United Kingdom had promoted a population health agenda in the general curriculum description of adult nursing programs. Expert consensus from Canada (46) and China (47) similarly highlighted insufficient systematic population health training within pre-registration nursing curricula. In accordance with these global educational deficiencies, the present study provided novel empirical evidence from the perspective of Chinese nursing students, further confirming the underdevelopment of population health education in domestic undergraduate nursing programs. Fewer than 20% of participants reported frequent access to teaching covering six core AACN population health competencies, demonstrating that systematic population health integration remains a major unresolved challenge for nursing education domestically and internationally.

In spite of students’ inadequate knowledge and limited educational exposure, it is encouraging that nearly half of nursing students held positive attitudes toward the necessity of population health competencies for clinical nurses and the value of integrating this content into undergraduate nursing training. Still, a notable proportion of students held opposing or neutral attitudes. Students aged 21 and older expressed significantly more supportive stances, implying older undergraduates may be more receptive to population health curriculum reforms. In addition, the primary reason respondents disagreed, strongly disagreed, or felt undecided about the necessity of population health training for undergraduate nursing students was their belief that “*population health is important only for entry-level nursing education, which aims to cultivate nursing practitioners or community nurses, but not for advanced-level nursing education that focuses on training clinical nurse specialists*.” This opinion contradicts Domain 3 of the AACN Essentials (36), which specifies that the population health domain and its core required competencies are identical across entry-level and advanced-level nursing education, with only tiered sub-competency expectations differing across practice levels. Importantly, despite their varying attitudes toward population health, 73.0% of students clearly expressed their willingness to receive structured population health instruction, laying a solid student-driven foundation for future curriculum integration initiatives.

When asked about their preferred implementation model for integrating population health content, the participating students expressed a preference for developing their population health competencies through “*lecture-based elective courses*” delivered *longitudinally* within the existing undergraduate nursing curriculum. To date, the teaching strategies utilized to foster population health competencies vary widely, and no standardized model of “best practices” has been established. U. S. nursing schools commonly adopt interactive case studies, group discussion, role-play, and student presentations to deliver relevant content (5). Community-focused educational projects can provide students with immersive and observational learning experiences, while didactic instruction is also recommended to strengthen students’ conceptual understanding of population health (22). The findings of the present study indicate that Chinese nursing students’ preference for elective lecture formats reflected a desire for flexible, optional access to population health coursework alongside mandatory clinical training modules.

Several study limitations must be acknowledged. First, the cross-sectional, single-center design and modest sample size may weaken the validity and generalizability of findings. Second, in terms of questionnaire development, several items (Q19 and Q22) adopted fixed pre-set response options, which might fail to capture students’ individualized and in-depth perspectives, thereby introducing potential measurement bias. Furthermore, the quantitative survey design cannot fully elucidate the underlying reasons and formative mechanisms of students’ attitudinal traits. Additionally, the evaluation of students’ perceived access to population health competency training was based on the 2021 AACN framework, which was originally developed for Western nursing education systems. Currently, no region-specific Asian standards or dedicated WHO guidelines are available for population health competency training in undergraduate nursing education. The adoption of this Western-centric framework may reduce contextual fit within China’s educational landscape. Moreover, the knowledge assessment in this study only examined students’ basic conceptual understanding of population health, covering its definition, core objectives, scope, and key subtopics, while students’ practical population health competencies were not quantitatively evaluated. In addition, the translated questionnaire only underwent content validity evaluation without formal cross-cultural adaptation or comprehensive psychometric testing, which may compromise instrument stability. Furthermore, a considerable participant attrition occurred during data collection. Voluntary participation, the 15-min time limit, and environmental distractions likely reduced the valid response rate, introducing mild sampling bias and further limiting cross-group inference. The narrow age distribution of participants also weakens the interpretability of age-stratified subgroup analyses. Future multi-center mixed-methods research with larger, more diverse samples is needed to replicate and extend these results. Localized population health competency evaluation tools tailored to China’s nursing education system should also be developed to support targeted curriculum improvement.

## 5 Conclusion

The present study revealed that nursing students surveyed at a Chinese university exhibited inadequate knowledge of population health and had limited exposure to undergraduate training aligned with the population health competencies outlined by the AACN. Nevertheless, participants generally held positive attitudes and intentions toward the integration of population health content into undergraduate nursing curricula. These results suggest that the sampled nursing students may be insufficiently prepared to fulfill core nursing responsibilities pertaining to public health prevention and population-based disease management across the healthcare continuum. Amid the global paradigm shift from individual-centric clinical care toward population-centered health systems, strengthening population health training has become an urgent priority for pre-registration nursing education. Nursing educators should expand competency-focused population health learning opportunities, particularly by designing a suite of “*lecture-based elective courses*” that are delivered *longitudinally* within the existing undergraduate nursing curriculum.

## Data Availability

The raw data supporting the conclusions of this article will be made available by the authors, without undue reservation.
