# Supplementary material for: Students’ knowledge, perceived access, and attitudes toward population health integration in undergraduate nursing education
Source: Front Public Health. 2026 Jul 17;14:1898158. doi: 10.3389/fpubh.2026.1898158 (PMC13424291; doi:10.3389/fpubh.2026.1898158)
Supplement: Supplementary file 1 [file Data_Sheet_1.pdf]

Supplementary Material

TRANSLATED QUESTIONNAIRE

1- Demographic Information.

Q1: Gender (Single choice) ○ Male ○ Female

Q2: Age ( ) years old.

Q3: Academic grade (Single choice)
○ 1 st-year undergraduate ○ 2 nd-year undergraduate
○ 3 rd-year undergraduate ○ 4 th-year undergraduate
○ Postgraduate

2- Population Health Knowledge.

Q4: Please self-evaluate your understanding of population health on a score of 1 to 5. (Single choice)
○ Very well understood (Score: 5) ○ Understood (Score: 4) ○ Unclear understanding (Score: 3)
○ Poorly understood (Score: 2) ○ Not understood at all (Score: 1)

Q5: Which of the following is the correct concept of population health? (Single choice)
○ Medical services and health resources provided by hospitals and community clinics.
○ Average life expectancy, mortality rate and their influencing factors in a specific country or region.
○ The health outcomes of a group of individuals, including the distribution of such outcomes within the group.
○ A discipline that targets populations and adopts methods such as epidemiology, statistics, behavioral science and clinical medicine to implement interventions before a disease occurs or during its subclinical stage, thereby interrupting the causal chain and reducing the risk of onset and death.
○ The art and science of preventing disease, prolonging life and promoting health for a specific population through the organized efforts of society.

Q6: Which of the following is NOT the core objective of population health? (Single choice)
○ Help provide evidence for and inform political judgments; address health and social determinants of health through health policy change.
○ Focus on treating patient groups with severe diseases and providing personalized clinical medical services for these groups.
○ Seeks to address upstream drivers of health inequities addressing factors of social cohesion and social capital within the socio-economic and political contexts.
○ Facilitate the development of strategies and public health programs and interventions based on preventive research, data analytics, and evidence-based practices

Q7: Which of the following is the correct definition of "population" in the context of population health? (Single choice)
○ All individuals who receive direct medical treatment from healthcare providers in a specific hospital or clinic.
○ A group of patients with the same disease who are under the care of a single medical team.
○ All residents living within a fixed geographic boundary, regardless of their health status or service needs.
○ A discrete group that the nurse and others care for across settings at local, regional, national, and global levels.
○ People who actively seek and utilize public health services within a certain period of time.

Q8: What is the relationship between population health and public health? (Single choice)
○ Population health and public health are two distinct yet interconnected disciplines.
○ As a broader concept, population health encompasses public health and other related fields.
○ Population health is synonymous with public health, serving as an alternative designation for the latter.
○ As a broader concept, public health encompasses population health and other related fields.
○ Population health and public health are two distinct disciplines, focusing on populations and individuals respectively.

Q9: Which of the following is NOT a subtopic of population health? (Single choice)
○ Public health prevention
○ Acute care
○ Ambulatory care
○ Long-term care
○ Collaborative activities among stakeholders – all relevant individuals and organizations involved in care, including patients and communities themselves
○ Incidence of outpatient visits and hospitalizations

3- Perceived Access to Population Health Education.

Please rate the frequency with which your undergraduate nursing curriculum provides opportunities to develop the following population health competencies (Q10-Q15). (Single choice for each item)

Q10: Manage population health
INCLUDING:
Assess the efficacy of a system's capability to serve a target sub-population's healthcare needs;
Analyze primary and secondary population health data for multiple populations against relevant benchmarks;
Use established or evolving methods to determine population-focused priorities for care;
Develop a collaborative approach with relevant stakeholders to address population healthcare needs, including evaluation methods;
Collaborate with appropriate stakeholders to implement a sociocultural and linguistically responsive intervention plan.

○ Never ○ Very infrequently ○ Occasionally ○ Often/frequently ○ Very frequently

Q11: Engage in effective partnerships
INCLUDING:
Ascertain collaborative opportunities for individuals and organizations to improve population health;
Challenge biases and barriers that impact population health outcomes;
Evaluate the effectiveness of partnerships for achieving health equity;
Lead partnerships to improve population health outcomes;
Assess preparation and readiness of partners to organize during natural and manmade disasters.

○ Never ○ Very infrequently ○ Occasionally ○ Often/frequently ○ Very frequently

Q12: Consider the socioeconomic impact of the delivery of health care
INCLUDING:
Analyze cost-benefits of selected populationbased interventions;
Collaborate with partners to secure and leverage resources necessary for effective, sustainable interventions;
Advocate for interventions that maximize costeffective, accessible, and equitable resources for populations;
Incorporate ethical principles in resource allocation in achieving equitable health.

○ Never ○ Very infrequently ○ Occasionally ○ Often/frequently ○ Very frequently

Q13: Advance equitable population health policy
INCLUDING:
Identify opportunities to influence the policy process;
Design comprehensive advocacy strategies to support the policy process;
Engage in strategies to influence policy change;
Contribute to policy development at the system, local, regional, or national levels;
Assess the impact of policy changes;
Evaluate the ability of policy to address disparities and inequities within segments of the population;
Evaluate the risks to population health associated with globalization.

○ Never ○ Very infrequently ○ Occasionally ○ Often/frequently ○ Very frequently

Q14: Demonstrate advocacy strategies
INCLUDING:
Appraise advocacy priorities for a population;
Strategize with an interdisciplinary group and others to develop effective advocacy approaches;
Engage in relationship-building activities with stakeholders at any level of influence, including system, local, state, national, and/or global;
Demonstrate leadership skills to promote advocacy efforts that include principles of social justice, diversity, equity, and inclusion.

○ Never ○ Very infrequently ○ Occasionally ○ Often/frequently ○ Very frequently

Q15: Advance preparedness to protect population health during disasters and public health emergencies
INCLUDING:
Collaboratively initiate rapid response activities to protect population health;
Participate in ethical decision making that includes diversity, equity, and inclusion in advanced preparedness to protect populations;
Collaborate with interdisciplinary teams to lead preparedness and mitigation efforts to protect population health with attention to the most vulnerable populations;
Coordinate the implementation of evidencebased infection control measures and proper use of personal protective equipment;
Contribute to system-level planning, decision making, and evaluation for disasters and public health emergencies.

○ Never ○ Very infrequently ○ Occasionally ○ Often/frequently ○ Very frequently

4- Attitudes Toward Population Health Integration in Undergraduate Nursing Curricula

Please indicate your level of agreement with the following statements (Q16-Q18,Q20) . (Single choice for each item)

Q16: It is necessary for clinical nurse specialists to possess population health competencies
○ Strongly disagree ○ Disagree ○ Neutral ○ Agree ○ Strongly agree

Q17: It is important to integrate population health into nursing education and training
○ Strongly disagree ○ Disagree ○ Neutral ○ Agree ○ Strongly agree

Q18: Population health training is necessary for undergraduate nursing students
○ Strongly disagree ○ Disagree ○ Neutral ○ Agree ○ Strongly agree

Q19: What is the most important reason for your disagreement with the necessity of population health training for undergraduate nursing students?(Single choice)
** Only respondents who selected "Disagree", "Strongly disagree", or "Undecided" for A3 need to answer this question.*
○ Population health is important only for entry-level nursing education, which aims to cultivate nursing practitioners or community nurses, but not for advanced-level nursing education that focuses on training clinical nurse specialists.
○ Population health should be incorporated into postgraduate nursing education, rather than into undergraduate nursing programs.
○ Population health is an independent discipline that is distinct from nursing science.

Q20: I really want to systematically learn about population health if it is successfully integrated into nursing education.
○ Strongly disagree ○ Disagree ○ Neutral ○ Agree ○ Strongly agree

Q21: What is your most preferred mode of integrating population health into undergraduate

nursing education? (Single choice)
○ Centralized. ○ Longitudinal distributed. ○ Multistage.

Q22: What is your most preferred pedagogical model for delivering content related to population health during undergraduate nursing education? (Single choice)**.**

○ Lecture-based elective course.
○ Small group discussion review of the literature.
○ Small group discussion of clinical cases.
○ Brief community experience.
○ Structured experience with a local healthcare organization.
○ Lecture-based required course.
○ Longitudinal community experience.
○ Dual degree in Nursing and Population Health.
○ Co-curricular activity.
